# Synthesis of ^64^Cu^II^–Bis(dithiocarbamatebisphosphonate) and Its Conjugation with Superparamagnetic Iron Oxide Nanoparticles: In Vivo Evaluation as Dual-Modality PET–MRI Agent**

**DOI:** 10.1002/anie.201007894

**Published:** 2011-05-04

**Authors:** Rafael Torres Martin de Rosales, Richard Tavaré, Rowena L Paul, Maite Jauregui-Osoro, Andrea Protti, Arnaud Glaria, Gopal Varma, Istvan Szanda, Philip J Blower

**Affiliations:** 1 Division of Imaging Sciences & Biomedical Engineering, King's College London4th Floor, Lambeth Wing, St. Thomas' Hospital, London SE1 7EH (UK)

**Keywords:** chelates, copper, imaging agents, nanoparticles, rare earths

The synergistic combination of positron emission tomography (PET) and magnetic resonance imaging (MRI) is likely to become the next generation of dual-modality scanners in medical imaging. These instruments will provide us with accurate diagnoses thanks to the sensitive and quantifiable signal of PET and the high soft-tissue resolution of MRI. Furthermore, patients will receive less radiation dose and spend less time in the procedure relative to current dual-modality scanners (e.g. PET–computed tomography (CT)). As a consequence, there has been increasing interest recently in the development of dual-modality PET–MRI agents.[1]

The majority of the PET–MRI agents reported to date are based on the combination of PET isotopes with superparamagnetic iron oxide (SPIO) nanoparticles.[2] These magnetic nanoparticles are ideal for the purpose, having a proven record of biocompatibility and a track record of extensive use in the clinic as MRI contrast agents for imaging the reticuloendothelial and lymphatic systems.[3] The radiolabeling of SPIOs has been done to date by often complicated chemical conjugation with their coatings, which are commonly biocompatible polymers such as dextran that provide them with colloidal stability. The polymeric coatings are typically bound relatively weakly to the surface of the SPIOs, which results in a lack of stability over time.[4] One solution to this problem is to cross-link the polymer units at the surface of the nanoparticles.[5] However, there are concerns for the translatability of these compounds due to toxic chemicals used in the synthesis.[4b]

**Figure 1:**
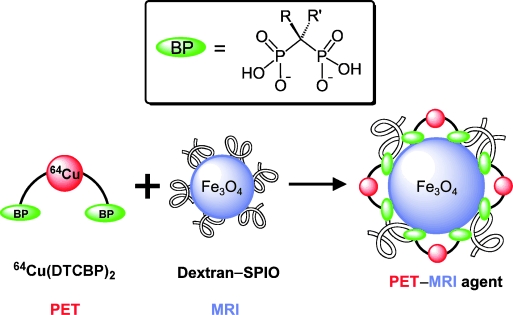
Schematic representations of a bisphosphonate (BP; top) and the conjugation reaction between the BP-based PET tracer [^64^Cu(dtcbp)_2_] and the dextran-coated iron oxide nanoparticle MRI probe Endorem/Feridex (bottom).

**Scheme 1:**
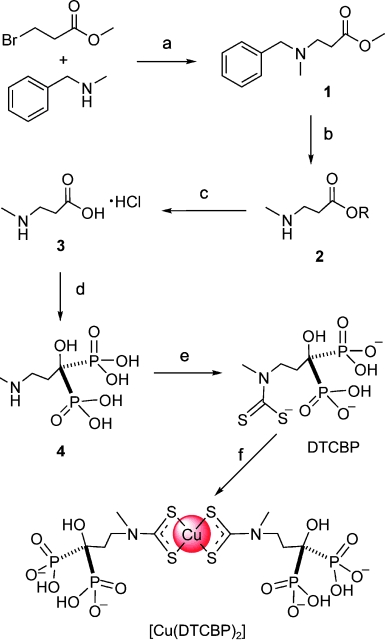
Synthesis of dtcbp and [Cu(dtcbp)_2_]. Reagents and conditions: a) Na_2_CO_3_ (10 equiv), CH_3_CN, 70 °C for 70 h; b) H_2_, 10 % Pd/C, EtOH, 48 h (R=Me, Et); c) 5 m HCl, reflux for 16 h; d) 1. Phosphorous acid (1.5 equiv), PCl_3_ (3.4 equiv), sulfolane, 67 °C for 3 h; 2. H_2_O, 100 °C for 1 h; e) CS_2_ (19 equiv), NaOH (7 equiv), THF, 24 h; f) 0.5 equiv Cu(OAc)_2_, H_2_O.

An alternative to radiolabeling the coatings of SPIO particles is to label their inorganic surface directly with a molecule that binds to both a PET isotope and the nanoparticle, leaving the polymeric coating unaffected. In this regard, we have recently reported that bisphosphonates (BPs; Figure 1) radiolabeled with a suitable isotope (^99m^Tc) for single photon emission computed tomography (SPECT) bind strongly to SPIO nanoparticles such as the dextran-coated MRI contrast agent Endorem/Feridex, and that the binding is stable in vitro and in vivo.[6] BPs hence have remarkable potential in the development of radionuclide-based SPIO imaging agents for dual-modality studies. Herein, we report the synthesis and characterization of a novel bifunctional BP conjugate that has been designed to bind to both SPIO nanoparticles for MR imaging and ^64^Cu for PET imaging. ^64^Cu is an isotope that is gaining attention for its favorable properties (half-life 12.7 h, 18 % β^+^, 39 % β^−^, 43 % electron capture) for PET and radionuclide therapy.[7] To bind ^64^Cu, we introduce the use of dithiocarbamate (dtc) as chelating group. The dtc group is a well-known ligand in coordination chemistry that binds to all transition metals,[8] including copper, but its use as a ^64^Cu chelator for PET imaging has been neglected.[9] The compound formed, [^64^Cu(dtcbp)_2_] (Scheme 1, Figure 1), has great affinity for iron oxide nanoparticles and other inorganic materials such as hydroxyapatite (HA) and rare-earth metal oxides such as gadolinium oxide (Gd_2_O_3_). Furthermore, we demonstrate that conjugation with clinically approved SPIOs gives nanoparticles that can be used for in vivo PET–MR lymphatic imaging (Figure 1).

Initial attempts to insert a dtc group into a BP were made by reaction of carbon disulfide with the amino group of alendronate, a primary amino-BP. The dtc–BP conjugate compound was formed and isolated in low yield, but lacked stability, readily decomposing at pH≤7 to release the starting materials. Dithiocarbamates derived from primary amines are known to be unstable under acid conditions. Therefore, we adopted the strategy of using a secondary amine instead. Monomethylation of amino-BPs was unfeasible because of the insolubility in organic solvents and high p*K*_a_ (ca. 12) of the amino groups of amino-BPs. A different synthetic strategy (Scheme 1) was chosen by combining a carboxylic acid for the formation of a BP and a methylated secondary amine separated by an ethylene spacer (**3**). The *N*-methylamino-BP (**4**) was then treated with CS_2_ to form the desired bifunctional chelator (dtcbp; Scheme 1).

**Figure 2:**
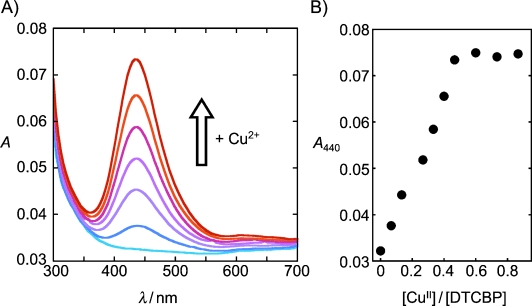
A) UV/Vis titration of dtcbp upon the addition of Cu^II^ ions (0–0.5 equiv) showing the increase in absorbance of the band at *λ*=440 nm due to the formation of [Cu(dtcbp)_2_]. B) Plot of absorbance at *λ*=440 nm against [Cu^II^]/[dtcbp] ratio. The absorbance increases until the [Cu^II^]/[dtcbp] ratio is 0.5, confirming the expected stoichiometry.

The ligand dtcbp has been designed to bind Cu ions through the dtc group to leave two BP groups free for binding to the surface of an iron oxide nanoparticle. A major concern in the design of dtcbp and other bifunctional metal chelators was whether Cu^II^ ions could coordinate to both the dtc and BP groups. Indeed, BPs have been reported to be good ligands for Cu.[10] Spectroscopic studies demonstrate, however, that dtcbp preferentially binds copper ions through its dtc group, and not the BP group. First, ESIMS studies of a solution of [Cu(dtcbp)_2_] demonstrate the presence and stoichiometry of the desired complex (ions observed: [*M*−2 H]^2−^, [*M*−3 H+Na]^2−^ and [*M*−4 H+2 Na]^2−^) (see the Supporting Information). Second, titration of Cu^II^ ions into a solution of dtcbp results in the appearance of an absorption band in the UV/Vis spectrum with *λ*_max_=440 nm, characteristic of square-planar Cu^II^–bis(dithiocarbamate) complexes (Figure 2).[8] The intensity of this band increases until 0.5 equivalents of Cu^II^ ions are present, which is consistent with the formation of the desired complex [Cu(dtcbp)_2_]. Furthermore, the data fit well to a 2:1 ligand/metal binding isotherm, which gives a value for log *K* of ≥10.1 (*K*=[ML_2_]/[M][L]^2^). The lack of involvement of the BP in copper binding is demonstrated by IR spectroscopy. Characteristic BP bands are observed in dtcbp at 954 and 999 cm^−1^ attributable to symmetrical and asymmetrical P–O vibrations, and at 1078 cm^−1^ for ν(P=O) vibrations.[11] The frequency of these vibrations remains unchanged after copper binding. ν(CS) vibrations usually found at around 1000 cm^−1^, which often provide information about the denticity of the DTC ligand, were not observed because of overlap with the intense BP bands. Copper binding, however, elicits a strong band at 1335 cm^−1^ owing to ν(N-CSS), suggesting a high degree of single bond character after metal complexation, as previously seen for other transition-metal–bis(DTC) complexes.[12]

**Figure 3:**
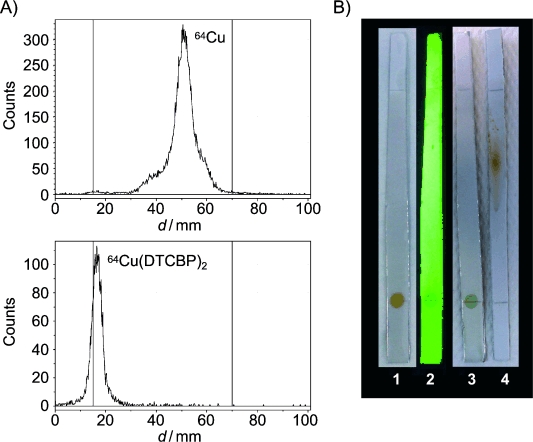
A) Radio-TLC chromatograms of free ^64^Cu (*R*_F_=0.66, top) and [^64^Cu(dtcbp)_2_] (*R*_F_=0.04, bottom). Vertical lines represent R_F_ values 0 (left) and 1 (right); B) Pictures of TLC plates showing: 1) [Cu(dtcbp)_2_] at *R*_F_=0.04 under white light; 2) the same TLC plate under UV light (*λ*=254 nm); 3) the same plate under white light after being stained with Dittmer–Lester’s reagent; 4) free Cu under white light, after being stained with a concentrated solution of diethyl dithiocarbamate. All TLC plates were made of silica gel and developed with 15 mm EDTA in 10 % NH_4_OAc/MeOH (50:50).

Radiolabeling of dtcbp with ^64^Cu to form [^64^Cu(dtcbp)_2_] was achieved by mixing an aliquot of a solution of ^64^Cu(OAc)_2_ in water with an aqueous solution of dtcbp in carbonate buffer at pH 9. As with the cold complex, the complexation proceeds instantaneously and quantitatively, and no heating is required. However, the high affinity and stability of the BP group to several inorganic materials (see below) made characterization particularly troublesome. Indeed, [^64^Cu(dtcbp)_2_] irreversibly binds to most chromatographic materials such as silica, silica-based reverse-phase (RP) (C18 and C8), polymer-based RP, and Al_2_O_3_ stationary phases when using common HPLC and TLC solvents, including ion-pairing conditions. Ion-exchange stationary phases also resulted in irreversible binding of the compound. Finally, radiolabeling yields were calculated using silica gel TLC with 15–50 mm ethylenediaminetetraacetic acid (EDTA) in 10 % NH_4_OAc/MeOH (50/50) as the mobile phase. Using this system, “free” ^64^Cu moves with a *R*_F_=0.66 (15 mm EDTA), whereas [^64^Cu(dtcbp)_2_] has a value of *R*_F_=0.04 (Figure 3 A). Very efficient labeling (10 GBq/mg, radiochemical yield=100 %) was found when dtcbp concentrations of ≥0.15 mm were used. To prove the chemical identity of [^64^Cu(dtcbp)_2_], the nonradioactive compound was analyzed using the same TLC method. Thus, [Cu(dtcbp)_2_] stays at the baseline of the TLC plate, which is in agreement with its radioactive analogue (Figure 3 B, images 1, 2, and 3). [Cu(dtcbp)_2_] can be seen by visible light owing to its absorbance at *λ*=440 nm (Figure 3 B (1)), which is characteristic of Cu^II^–bis(dithiocarbamate) ligand to metal charge transfer (LMCT) transitions, and it is UV-active at *λ*=254 nm (Figure 3 B (2)). Furthermore, the spot becomes light green after staining the TLC plate with Dittmer–Lester’s reagent, indicating the presence of phosphorus (Figure 3 B (3)). Free, nonradioactive Cu, on the other hand, migrates with *R*_F_=0.66, as found for ^64^Cu (Figure 3 B (4)).

The stability of [^64^Cu(dtcbp)_2_] was confirmed in phosphate-buffered saline (PBS) and human serum for at least 48 h. Incubation at 37 °C in these media showed no decomposition during this time using the TLC method described above. Furthermore, the complex does not decompose under the TLC conditions used (up to 50 mm EDTA), thus demonstrating high inertness towards ligand substitution. Subjecting the complex to more challenging conditions such as incubation in 3 mm EDTA solution at pH 4 results in partial decomposition only after 5 h. To determine if [^64^Cu(dtcbp)_2_] binds to serum proteins, these were precipitated by addition of ethanol. Thus, in serum, [^64^Cu(dtcbp)_2_] appears to bind completely to proteins. However, the binding was reversed if an insoluble material with known affinity towards BPs, such as HA, was added to the serum–[^64^Cu(dtcbp)_2_] mixture at various time points within 48 h. This resulted in complete binding of [^64^Cu(dtcbp)_2_] to HA, suggesting that the binding to serum proteins is weak and that the complex is inert to transchelation by copper-binding biomolecules present in human serum.

**Figure 4:**
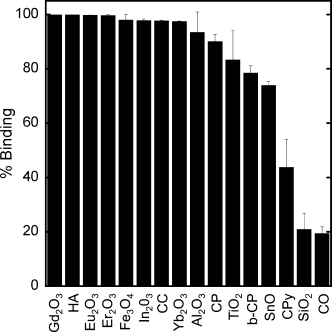
In vitro binding study of [^64^Cu(dtcbp)_2_] in 50 mm tris(hydroxymethyl)aminomethane pH 7 at room temperature to various inorganic materials (1 mg mL^−1^) after 1 h incubation. Abbreviations: hydroxyapatite (HA); calcium carbonate (CC); calcium phosphate (CP); β-tricalcium phosphate (b-CP); calcium pyrophosphate (CPy), and calcium oxalate (CO).

BPs are well-known strong binders of several inorganic materials, including calcium salts such as HA, and metal oxides such as TiO_2_, ZrO_2_, SiO_2_, and Fe_3_O_4_.[13] Indeed, we tested the binding of [^64^Cu(dtcbp)_2_] to several of these salts showing high binding (≥97 %) to HA, Fe_3_O_4_, and calcium carbonate (CC; Figure 4). Interestingly, [^64^Cu(dtcbp)_2_] also binds to rare-earth metal oxides of the type M_2_O_3_ (M=Gd, Er, Eu, Yb). It is also worth noting that the presence of two BP moieties in [^64^Cu(dtcbp)_2_] increases the binding capabilities to these materials when compared to mono-BP compounds, which seem to be selective for HA among the calcium salts.[14] [^64^Cu(dtcbp)_2_], however, binds well to HA, CC, calcium phosphate (CP), and *β*-tricalcium phosphate (b-CP).

The affinity of [^64^Cu(dtcbp)_2_] towards Fe_3_O_4_ allows us to demonstrate the potential of this compound for the synthesis of dual-modality PET–MR imaging agents. Labeling of clinically available SPIO nanoparticles (Endorem/Feridex) with [^64^Cu(dtcbp)_2_] was performed as follows: Endorem solution (15 μL) was added to a solution of [^64^Cu(dtcbp)_2_], and the mixture was heated at 100 °C for 15 min. This step is necessary to achieve maximum radiochemical yields (presumably by assisting the BP groups to permeate the loosely bound dextran coating and bind to the iron oxide surface of the nanoparticles). Limiting the heating period to 15 min maintains the colloidal stability of the solution.[6] The radiolabeled nanoparticles (hydrodynamic size=(108±60) nm) were then purified by centrifugal filtration of the colloidal solution with a 10 kDa molecular weight cut-off (MWCO) membrane, which removes unbound [^64^Cu(dtcbp)_2_]. Radiolabeling yields of 95 % were obtained (100 % radiochemical purity). The stability of the nanoparticle–BP interaction was studied for [^64^Cu(dtcbp)_2_]–Endorem in PBS and human serum by separating the nanoparticles from the media using centrifugation and 100 kDa MWCO filters at several time points. [^64^Cu(dtcbp)_2_] remained bound quantitatively to the magnetic nanoparticles in both media at 37 °C for at least 48 h. We also studied the stability of [^64^Cu(dtcbp)_2_]–Endorem in high concentrations of EDTA (10 mm) at pH 4, showing that ^64^Cu remains associated with Endorem for at least 24 h, which is in contrast to [^64^Cu(dtcbp)_2_], for which extensive decomposition is evident within 5 h. Thus, it seems that conjugation to the nanoparticles or the protective effect of the dextran polymer coating prevents transchelation in vitro.

**Figure 5:**
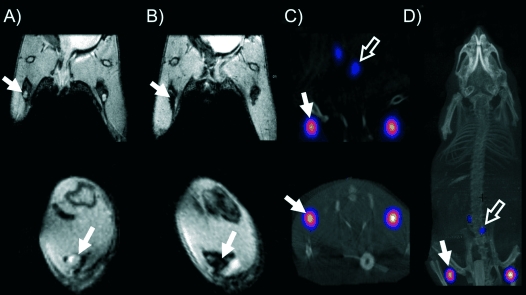
In vivo PET–MR imaging studies with [^64^Cu(dtcbp)_2_]–Endorem in a mouse. A,B) Coronal (top) and short axis (bottom) MR images of the lower abdominal area and upper hind legs showing the popliteal lymph nodes (solid arrows) before (A) and after (B) footpad injection of [^64^Cu(dtcbp)_2_]–Endorem. C) Coronal (top) and short-axis (bottom) NanoPET–CT images of the same mouse as in (B) showing the uptake of [^64^Cu(dtcbp)_2_]–Endorem in the popliteal (solid arrow) and iliac lymph nodes (hollow arrow). D) Whole-body NanoPET–CT images showing sole uptake of [^64^Cu(dtcbp)_2_]–Endorem in the popliteal and iliac lymph nodes. No translocation of radioactivity to other tissues was detected.

In vivo PET–MR imaging studies with [^64^Cu(dtcbp)_2_]–Endorem were carried out sequentially in a 9.4 T NMR magnet and a NanoPET–CT scanner (Figure 5). The lymphatic system was chosen as in vivo model because of the clinical need for accurate quantification of lymph node uptake using imaging, especially in oncologic studies in which the uptake of SPIO nanoparticles and ^99m^Tc colloids in sentinel lymph nodes have been shown to provide a measure of cancer spread.[15] First, *T*_2_*-weighted MR images of the lower abdominal area and legs of an anaesthetized C57BL/6 mouse were obtained, and the popliteal lymph nodes were located (Figure 5 A, solid arrows). The mouse was then injected in the footpads with 2 MBq (20 μL, 44 μg Fe) [^64^Cu(dtcbp)_2_]–Endorem. After 3 h, the animal was imaged again using the same parameters and showed significant decrease in signal and hence accumulation of Endorem in the popliteal lymph nodes (Figure 5 B). The mouse was then transferred to the NanoPET–CT scanner and an image acquired. Uptake in the popliteal lymph nodes and, to a lesser extent, in the iliac lymph nodes was observed (Figure 5 C,D), confirming co-location of ^64^Cu and Endorem in draining lymph nodes.

In summary, we have described the design, synthesis, and characterization of dtcbp, a novel bifunctional chelator containing a dithiocarbamate group for binding the PET isotope ^64^Cu, and a BP group for strong binding to Fe_3_O_4_ and other inorganic materials, such as HA and rare-earth oxides. The ligand dtcbp binds ^64^Cu efficiently to form Cu^II^–bis(dithiocarbamatebisphosphonate), and the complex is stable in vitro for at least 2 days. [^64^Cu(dtcbp)_2_] is not as thermodynamically stable or kinetically inert under highly acidic conditions or in the presence of high concentrations of EDTA as complexes derived from macrocyclic ligands (*t*_1/2_<5 min in 5 m HCl at 90 °C compared to 154 h for Cu-CB-TE2 A),[7, 16] but is sufficiently inert to metal transchelation while retaining the advantage of fast metal-binding kinetics at room temperature. This is an important factor when radiolabeling temperature-sensitive compounds. [^64^Cu(dtcbp)_2_] binds several inorganic materials with high affinity, including Endorem/Feridex and several rare-earth metal oxides that have promising MR contrast properties, such as Gd_2_O_3_, or luminescent properties, such as Eu_2_O_3_. The PET–MR dual-modality imaging capabilities of [^64^Cu(dtcbp)_2_]–Endorem were demonstrated in vivo by showing that it accumulates in draining lymph nodes. [^64^Cu(dtcbp)_2_]–Endorem should allow easy and accurate quantification of its uptake in vivo using the PET–MR instrumentation currently in development.[17] Radiolabeling of iron or rare-earth oxide materials with [^64^Cu(dtcbp)_2_] and other BP-based radiotracers in combination with BP-targeting/stability molecules could be used as a clean and simple method to synthesize targeted PET–MR or PET–optical-imaging agents.
