# Supplementary material for: Synthesis of 64CuII–Bis(dithiocarbamatebisphosphonate) and Its Conjugation with Superparamagnetic Iron Oxide Nanoparticles: In Vivo Evaluation as Dual-Modality PET–MRI Agent
Source: Angew Chem Int Ed Engl. 2011 May 4;50(24):5509–13. doi: 10.1002/anie.201007894 (PMC3179845; doi:10.1002/anie.201007894)
Supplement: Supplementary file 1 [file anie0050-5509-SD1.pdf]

Angewandte Chemie
Eine Zeitschrift der Gesellschaft Deutscher Chemiker

Supporting Information
© Wiley-VCH 2011
69451 Weinheim, Germany

Synthesis of $^{64}Cu^{II}$–Bis(dithiocarbamatebisphosphonate) and Its Conjugation with Superparamagnetic Iron Oxide Nanoparticles: In Vivo Evaluation as Dual-Modality PET–MRI Agent**

Rafael Torres Martin de Rosales, Richard Tavaré, Rowena L. Paul, Maite Jauregui-Osoro, Andrea Protti, Arnaud Glaria, Gopal Varma, Istvan Szanda, and Philip J. Blower**

anie_201007894_sm_miscellaneous_information.pdf

Experimental details

Materials and instrumentation. Reagents and materials were obtained from commercial sources and used as received unless otherwise noted. Fine Chemicals were obtained from Sigma Chemical Co. (Poole, UK). ^{64}Ni (99.6 % isotopically enriched) was purchased from CK GAS products Ltd. (Hampshire, UK). Organic solvents were of HPLC grade. Water (Type I, 18.2 MΩ·cm) was obtained from an ELGA Purelab Option-Q system. Endorem was obtained from Guerbet GmbH (France) and used as received. Human serum from human male AB plasma was obtained from Sigma-Aldrich. For TLC studies, 0.5 × 10 cm silica gel 60 F-254 aluminum backed plates (Merck KGaA, Germany) were used with spotting line at 1.5 cm and solvent front at 7 cm, using 15-50 mM EDTA in 10% Ammonium acetate/MeOH (50/50) (v/v) as the mobile phase. Dittmer-Lester's TLC reagent for the detection of phosphorus was prepared following the original literature protocol.[1] TLC plates were scanned with a Mini-Scan TLC Scanner equipped with a FC3600 detector optimized for the detection of β^+ particles (Lablogic, UK). Radioactivity in samples was measured with a CRC-25R dose calibrator (Capintec, USA) or a 1282 CompuGamma gamma counter (LKB Wallac, Finland). Radiolabeled nanoparticles were purified using Vivaspin 500 filters with a 10 kDa molecular weight cut-off (GE Healthcare, UK). Millex IC 0.22 μm (13 mm) hydrophilic PTFE filters (Millipore, USA) were used throughout this study. High-resolution mass spectra (HR-MS) were obtained at the Division of Imaging Sciences using an Agilent 6500 Accurate-Mass Q-TOF LC-MS system using electrospray ionization. HR-MS of $Cu(DTCBP)_2$ was obtained at the EPSRC National Mass Spectrometry Service Centre at Swansea University using a Thermo Scientific LTQ Orbitrap XL spectrometer coupled to an

Advion TriVersa NanoMate using nanoelectrospray ionization in the negative mode. NMR spectra were obtained in a 400 MHz Bruker Avance III (Germany). ^{1}H chemical shifts are referenced with respect to the residual solvent peak (δ_H 4.79 ppm, D_2O; 7.26 ppm $CDCl_3$).[2] ^{31}P resonances were referenced to an external solution of 85% H_3PO_4 (δ_P 0 ppm). ^{13}C chemical shifts were referenced to the residual solvent peak (δ_C 77.16 ppm, $CDCl_3$) or left unreferenced (D_2O). Elemental analyses were carried out at the Elemental Analysis Service at London Metropolitan University, UK. Dynamic Light Scattering (DLS) measurements were performed with a Beckman Coulter DelsaNano C instrument at 25°C. TEM analyses were performed in a FEI Tecnai T20 instrument with a LaB_6 filament operating at 200 kV. The samples were prepared by evaporation of a drop of the aqueous colloidal suspensions onto a carbon-coated copper grid (Agar Scientific 200 mesh). Iron oxide (as magnetite nanoparticles) lacking coating molecules used for the in vitro binding study was synthesized following an established method by Stroeve *et al.*[3] IR spectra were obtained in a Perkin-Elmer Spectrum 100 spectrometer equipped with a universal ATR sampling accessory.

Production of ^{64}Cu. ^{64}Cu was produced at the PET Imaging Centre, St. Thomas' Hospital, London, UK by proton bombardment of 10-15 mg of ^{64}Ni, plated onto a gold disc, at 25 μA for 4-6 hours in a CTI RDS 112 11 MeV cyclotron using purpose-built targetry. Purification of ^{64}Cu was undertaken using established procedures.[4] Radionuclidic purity was confirmed by gamma-ray spectroscopy (Ortec DSPEC Plus HPGe gamma-ray spectrometer) to be >99 %. $^{64}CuCl_2$ was then obtained in HCl solution and was transformed into $^{64}Cu(OAc)_2$ by evaporation of the solution to a small volume

and titrating it with small aliquots of a 1 M sodium acetate solution (99.995 % trace metal basis) until the pH of the solution reached 6. Purity was confirmed by TLC (R_F = 0.66) with silica gel plates using 15 mM EDTA in 10% Ammonium acetate/MeOH (50/50) as the mobile phase.

Synthesis of DTCBP.

Methyl 3-(benzyl(methyl)amino)propanoate (1). Methyl 3-bromopropanoate (5.00 g, 29.9 mmol) and *N*-methyl-1-benzylamine (3.63 g, 29.9 mmol) were dissolved in acetonitrile (200 mL). After 2 min. stirring at room temperature, Na_2CO_3 (31 g, 299 mmol) was added and the temperature increased to 70 ºC for 70 h. After cooling, the reaction solution was filtered, and to the filtrate were added 100 mL of 2M NaOH. The product was extracted with 3 × 100 mL of CH_2Cl_2 and the extracts dried over Na_2SO_4. After filtration, the solvents were evaporated under vacuum to yield the product as a colourless oil (5.89 g, 95 %).

^{1}H-NMR ($CDCl_3$, 400.3 MHz, 298 K) δ_H (ppm) 7.34 (m, 5H, C_6H_5-CH_2-N), 3.70 (s, 3H, -COOCH_3), 3.54 (s, 2H, C_6H_5-CH_2-N), 2.77 (t, J = 7.6 Hz, 2H, -N-CH_2-CH_2-), 2.55 (t, J = 7.6 Hz, 2H, -CH_2-$COOCH_3$), 2.23 (s, 3H, -N-CH_3); ^{13}C-NMR ($CDCl_3$, 100.7 MHz, 298 K) δ_C (ppm) 173.03 (-$C$$OOCH_3$), 138.88, 128.91, 128.20 and 127.03 (C_6H_5-CH_2-), 62.11 (C_6H_5-$C$$H_2$-), 52.74 (-N-$C$$H_2$-$CH_2$-), 51.56 (-COO$C$$H_3$), 41.91 (-N-$C$$H_3$), 32.75 (-N-$CH_2$-$C$$H_2$-). HR-MS (ESI) 208.1339 (M+H$^+$, found), 208.1338 (M+H$^+$, calculated). Elemental analysis, found: C, 69.57; H, 8.18; N, 6.63. $C_{12}H_{17}NO_2$ requires: C, 69.54; H, 8.27; N, 6.76.

3-(Methylamino)propanoic acid hydrochloride (3). A schlenk tube was charged with methyl 3-(benzyl(methyl)amino)propanoate (1) (2 g, 9.64 mmol) and dissolved in EtOH (60 mL). To this solution were added 500 mg of 10% Pd/C in small portions. The atmosphere of the tube was purged first with oxygen-free N_2 followed by H_2. A double-layer latex balloon filled with H_2 (~ 1 L) was then attached to the Schlenk tube and the reaction was left stirring at room temperature for 48 h, during which the balloon was refilled as needed with H_2. At this point, the flask was opened and Celite added to the reaction mixture followed by filtration with Celite. The filtrate was evaporated to dryness to yield a yellowish oil. ^{1}H- and ^{13}C-NMR confirmed complete amine deprotection. However, it was also noted that transesterification had occurred to some extent. The crude product (2, Figure 1) was used for the next step without modification. Thus, the mixture of methyl and ethyl esters was dissolved in 40 mL of 5 M HCl and refluxed for 16 h. The solvent was then evaporated under vacuum to yield the product as a hygroscopic white solid (0.969 g, 72 %).

^{1}H-NMR (D_2O, 400.3 MHz, 298 K) δ_H (ppm) 3.33 (t, J = 6.4 Hz, 2H, -N-CH_2-CH$_2$-), 2.85 (t, J = 6.4 Hz, 2H, -CH_2-COOH), 2.76 (s, 3H, -N-CH_3); ^{13}C-NMR (D_2O, 100.7 MHz, 298 K) δ_C (ppm) 174.32 (-*C*OOH), 44.56 (-NH-*C*H$_2$-), 33.08 (-CH$_2$-*C*H$_2$-), 30.05 (-NH-*C*H$_3$). HR-MS (ESI) 104.0711 (M+H$^+$, found), 104.0706 (M+H$^+$, calculated). Elemental analysis, found: C, 34.37; H, 7.35; N, 9.88. $C_4H_{10}ClNO_2$ requires: C, 34.42; H, 7.22; N, 10.03.

1-Hydroxy-3-(methylamino)propane-1,1-diphosphonic acid hydrochloride (4). 3-(Methylamino)propanoic acid hydrochloride (3) (0.969 g, 6.9 mmol) and phosphorous acid (0.849 g, 10.4 mmol) were suspended in 3.5 mL of sulfolane and heated to 75 ºC for 30 min, resulting in the complete solution of both reagents. The mixture was then cooled to 35 ºC and PCl_3 (3.22 g, 23.4 mmol), added dropwise over 5 min. followed by heating at 67 ºC for 3 h. At this point, the reaction solution was cooled to 0 ºC and 10 mL of H_2O were added dropwise over 5 mins. Charcoal was added to the flask and the mixture was then refluxed for 1 h., filtered through celite and cooled to 0 ºC. To this solution was added EtOH until a white precipitate appeared. After 12 h standing at 4 ºC, an oily residue had separated. The residue was washed with ethanol and recrystallized from EtOH/H_2O at 4 ºC to yield the pure product as a white solid (781 mg, 39%).

^{1}H-NMR (D_2O, 400.3 MHz, 298 K) δ_H (ppm) 3.39 (t, J = 6.6 Hz, 2H, -N-CH_2-CH_2-), 2.73 (s, 3H, -N-CH_3), 2.85 (m, 2H, -CH_2-C(OH)(PO_3H_2)$_2$); ^{13}C-NMR (D_2O, 100.7 MHz, 298 K) δ_C (ppm) 72.16 (t, $^{1}J_{C\text{-}P}$ = 140 Hz, -*C*(OH)(PO_3H_2)$_2$), 45.59 (-NH-*C*H_2-), 32.93 (-NH-*C*H_3), 29.40 (-CH_2-*C*H_2-); ^{31}P{^{1}H} NMR (D_2O, 162.1 MHz, 298 K): δ_P (ppm); 17.47. HR-MS (ESI): 248.0103 ([M-H]$^-$, found), 248.0094 ([M-H]$^-$, calculated). Elemental analysis, found: C, 16.91; H, 4.95; N, 4.82. $C_4H_{14}ClNO_7P_2$ requires: C, 16.82; H, 4.94; N, 4.91.

Pentasodium mono(3-hydroxy-3,3-diphosphonatopropyl(methyl)dithiocarbamate) (DTCBP). 1-Hydroxy-3-(methylamino)propane-1,1-diphosphonic acid hydrochloride (4) (70 mg, 0.25 mmol) was suspended in 10 mL THF. To this suspension was added NaOH

(69 mg, 1.75 mmol, 7 eq) dissolved in 0.3 mL H_2O, followed by 0.7 mL of H_2O. The clear solution had a pH of 12. The solution was cooled to 0 ºC and CS_2 (373 mg, 4.9 mmol, 19.6 eq), dissolved in 1 mL of THF, was slowly added over 5 min. The reaction solution became cloudy. After 24 h stirring at room temperature the pH had dropped to 10 and the color had changed from colorless to faint yellow. At this point the excess CS_2 and THF were evaporated using a rotary evaporator and 20 mL of acetone were added to the mixture, resulting in the separation of an oil after storage at 4 ºC overnight. The oil was isolated by decantation of the supernatant, washed with 5 mL of acetone, dissolved in H_2O and lyophilized to yield the product as a hygroscopic white crystalline powder (120 mg, 77 %).

^{1}H-NMR (D_2O, 400.3 MHz, 298 K) δ_H (ppm) 4.34 (t, J = 7.7 Hz, 2H, -CH_2-N-CS_2), 3.49 (s, 3H, -N-CH_3), 2.24 (m, 2H, -CH_2-C(OH)(PO_3)$_2$); ^{13}C-NMR (D_2O, 100.7 MHz, 298 K) δ_C (ppm) 206.2 (-CS_2), 74.85 (t, $^1J_{C\text{-}P}$ = 140 Hz, -*C*(OH)(PO_3H_2)$_2$), 53.93 (-N-*C*H_2-), 42.98 (-N-*C*H_3), 31.92 (-CH_2-*C*H_2-); ^{31}P{^{1}H}naud NMR (D_2O, 162.1 MHz, 298 K): δ_P (ppm); 18.03. MS (ESI); 161.2 ($[M-2H]^{2-}$, found), 161.5 ($[M-2H]^{2-}$, calculated); 172.5 ($[M-3H+Na]^{2-}$, found), 172.5 ($[M-3H+Na]^{2-}$, calculated); 323.9 ($[M-H]^-$, found), 324.0 ($[M-H]^-$, calculated); 346.0 ($[M-2H+Na]^-$ found), 345.9 (($[M-2H+Na]^-$, calculated); 368.0 ($[M-3H+Na]^-$ found), 367.9 (($[M-3H+Na]^-$, calculated). ATR-IR (cm^{-1}): 3305 (br), 1639 (m), 1486 (w), 1383 (vw), 1252 (vw), 1078 (vs), 999 (s), 955(s). Elemental analysis, found: C, 9.63; H, 3.20; N, 1.96. $(C_5H_8NNa_5O_7P_2S_2)\cdot(NaCl)_{1.5}(H_2O)_{10}$ requires: C, 9.66; H, 3.08; N, 2.25.

Synthesis of Cu(II)(DTCBP)$_2$. DTCBP (13 mg, 1.85×10^{-2} mmol) was dissolved in 100 μL of 50 mM carbonate buffer (pH 9) and then $CuCl_2$ (0.5 eq, 1.2 mg) was added, resulting in the solution turning to a dark-brown color. After 5 minutes stirring at room temperature, the solution was freeze-dried to yield the product as a dark-brown powder.

HR-MS(ESI) (Figs. S1-S3): $[Cu(II)(DTCBP)_2]^{2-}$ (100%, 354.4105 m/z); $Na[Cu(II)(DTCBP)_2]^{2-}$ (80%, 365.4013 m/z) and $Na_2[Cu(II)(DTCBP)_2]^{2-}$ (50%, 376.3922 m/z). ATR-IR (cm^{-1}): 3275 (br), 1661(m), 1621(m), 1524(vw), 1335 (s), 1077 (vs), 996 (s), 956(s).

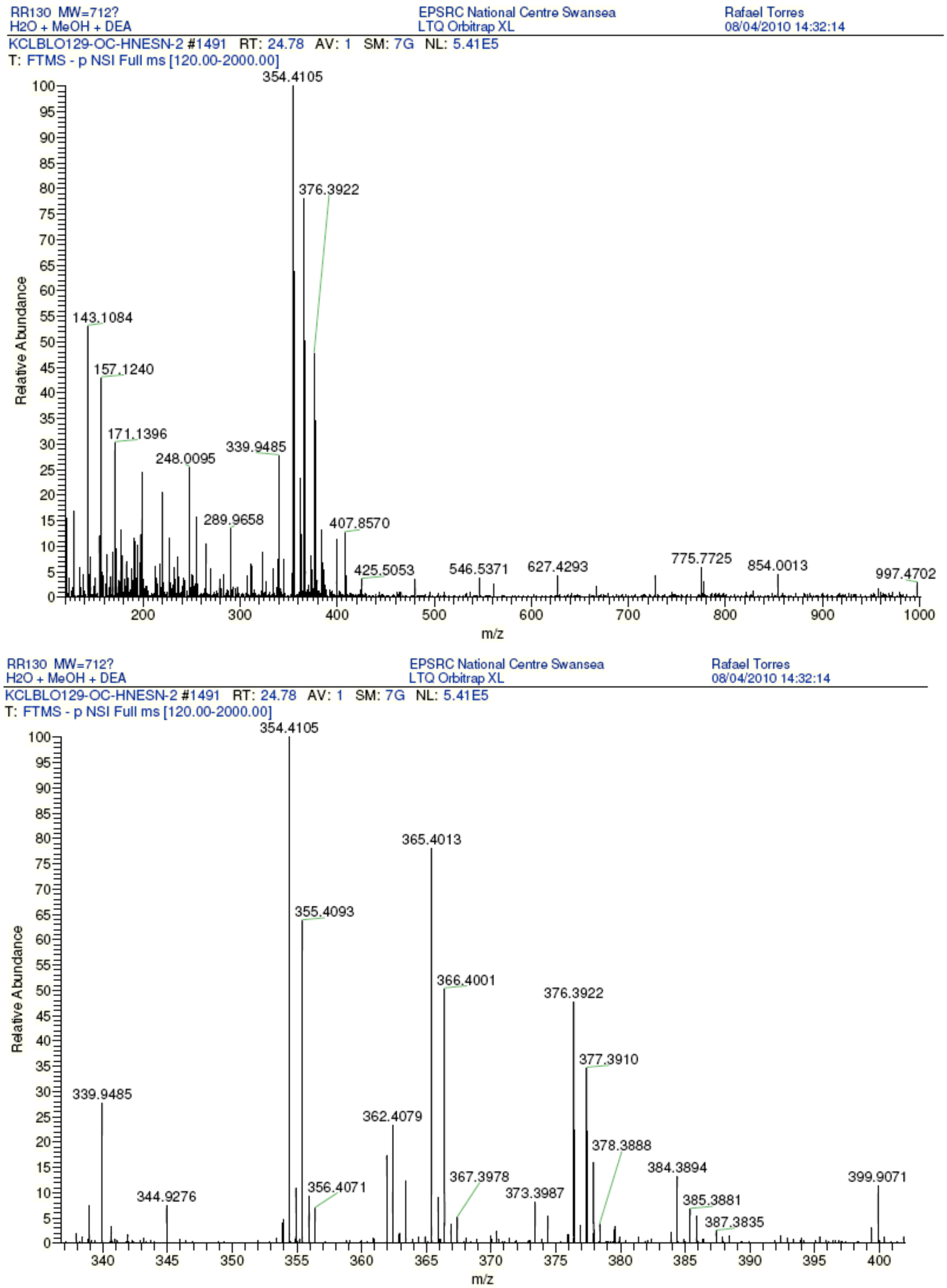

Fig S1. Full HR-ESI-MS spectra of a solution of $Cu(DTCBP)_2$ showing the following ions: ($[Cu(DTCBP)_2]^{2-}$ (100%, 354.4105 m/z), $Na[Cu(DTCBP)_2]^{2-}$ (80%, 365.4013 m/z) and $Na_2[Cu(DTCBP)_2]^{2-}$ (50%, 376.3922 m/z)

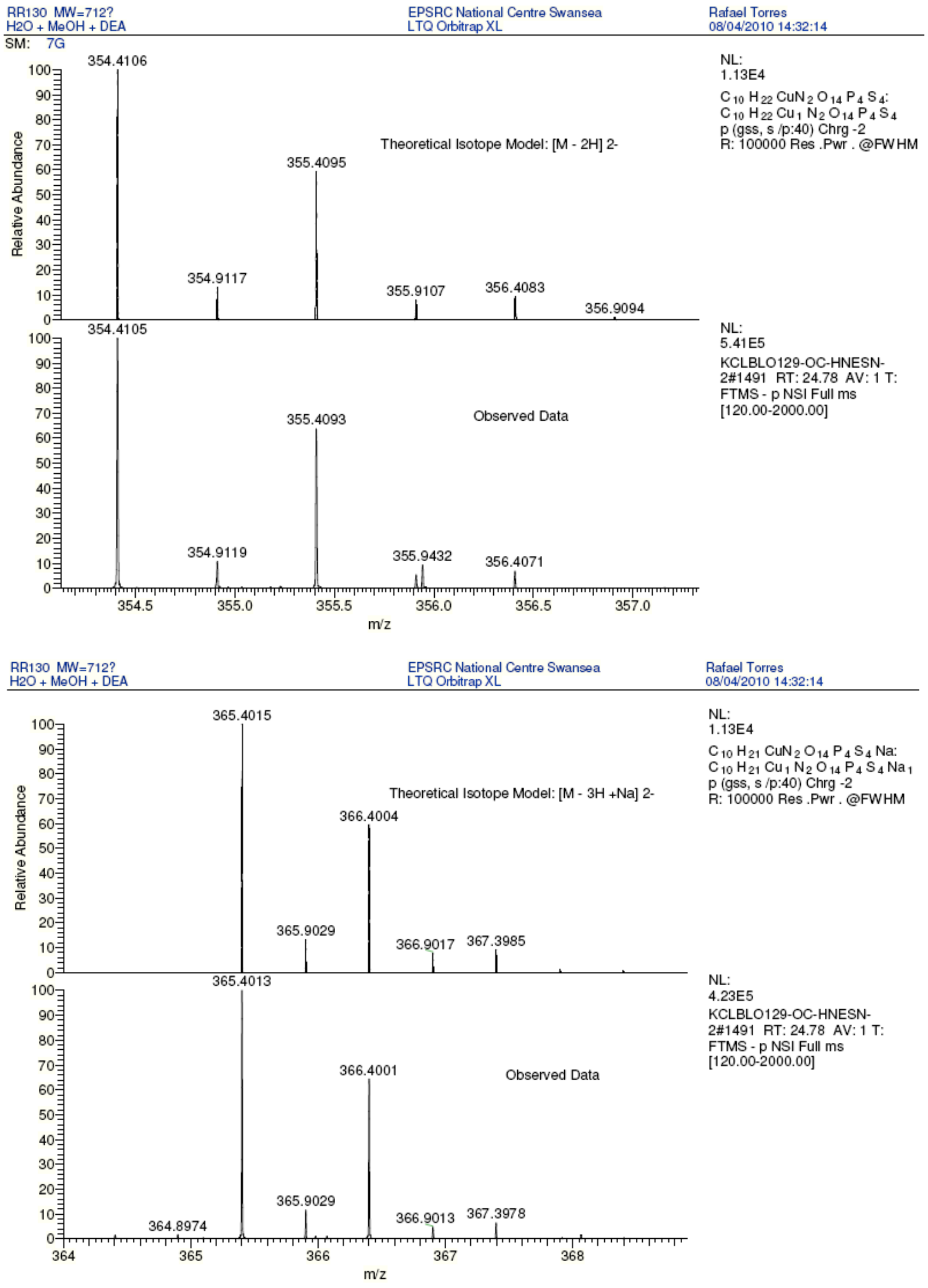

Fig S2. Theoretical and observed HR-ESI-MS specta of the ions: $[Cu(DTCBP)_2]^{2-}$ (top, 354.4105 m/z) and $Na[Cu(DTCBP)_2]^{2-}$ (bottom, 365.4013 m/z).

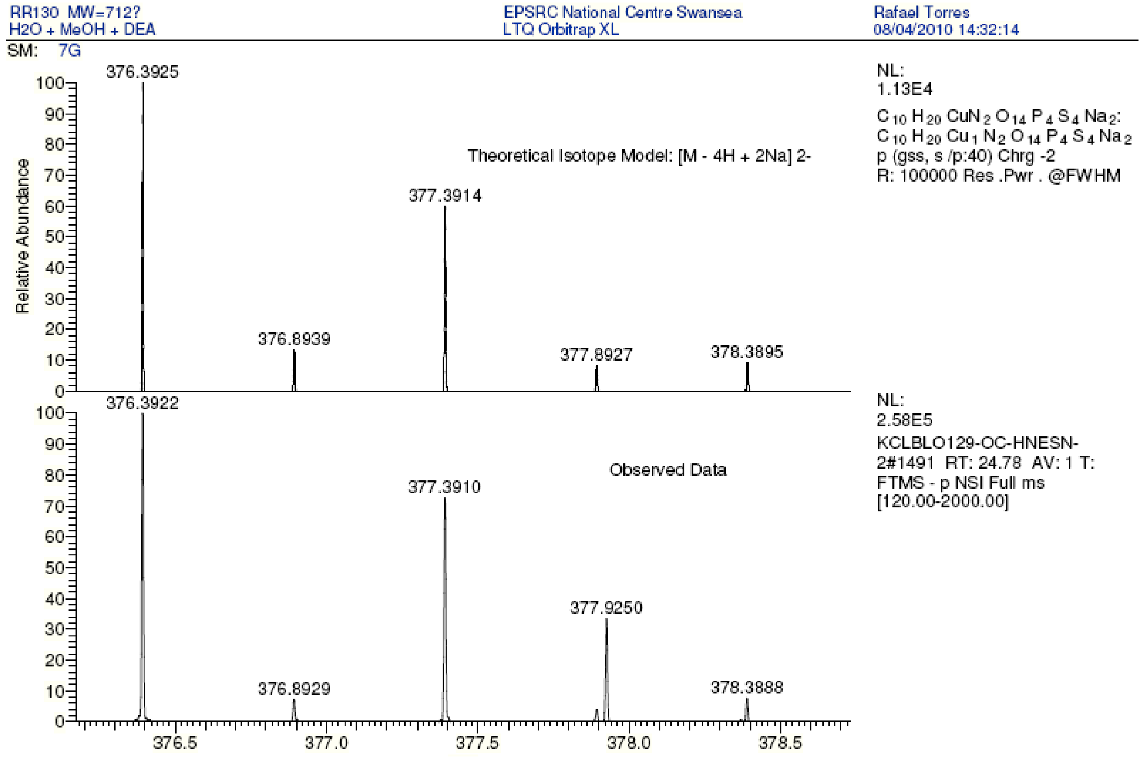

Fig S3. Theoretical (top) and observed HR-ESI-MS spectrum of the ion $Na_2[Cu(DTCBP)_2]^{2-}$ (376.3922 m/z)

Cu(II) titration and binding constant calculation: The binding constant of Cu(II) to DTCBP was measured by UV-vis spectroscopy. Aliquots of a $CuCl_2$ stock solution in water were added to of a 3.3×10^{-8} M solution of DTCBP in 50 mM carbonate buffer (pH 9). Upon addition of the Cu(II) ions to the DTCBP solution, an absorption band with λ_{max}= 440 nm appeared in the spectrum and its intensity increased until 0.5 eq of Cu(II) had been added. Addition of increasing amounts of Cu(II) did not result in any changes in the UV-vis spectrum until 1 equiv were present, when a bright green precipitate formed resulting in scattering. The green precipitate was analyzed by IR spectroscopy and was confirmed to be insoluble copper(II) carbonate.

The dissociation constant and the stoichiometry of the interaction for the equilibrium:

$$(1/n)\ [Cu(DTCBP)_n] \Leftrightarrow DTCBP + (1/n)\ Cu$$

were obtained by fitting the observed absorbance data at 440 nm to the following isotherm following a published protocol:[5]

$$y = \frac{\left(\dfrac{K^n \times x}{n}\right)}{\left(C - C \times M0\right)^n} + \frac{x}{n}$$

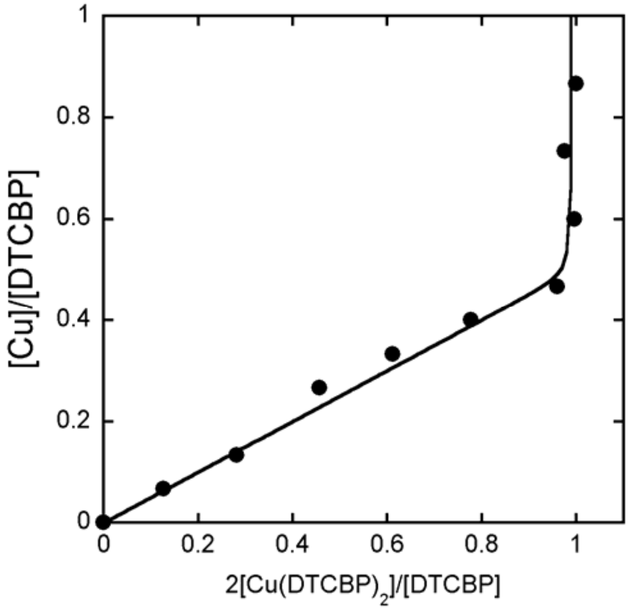

Where y = [Cu]/[DTCBP]; x = Saturation fraction = 2[Cu(DTCBP)$_2$]/[DTCBP] = (A – A_o)/($A_{¥}$ – A_o) where A_o, A, and $A_{¥}$ are the absorbance at 440 nm in the absence, in the presence, and at saturation of copper ions; C = [DTCBP]$_{total}$; n = 2; $K = K_d$ =

$[Cu][DTCBP]^2/[Cu(DTCBP)_2] = 8 \times 10^{-11}$. This corresponds to an association constant $K_a = 1.25 \times 10^{10}$ and a log $K = 10.1$.

Radiosynthesis of $^{64}Cu(II)(DTCBP)_2$. To establish the minimum concentration at which DTCBP could be efficiently labeled with ^{64}Cu, four solutions of different concentrations of DTCBP were prepared (1.5×10^{-3} M, 1.5×10^{-4} M, 1.7×10^{-6} M and 1.7×10^{-8} M) in 100 mM carbonate buffer at pH 9. An aliquot (100 μL) of each solution was mixed with 100 μL of a $^{64}Cu(OAc)_2$ solution at pH ≥ 7 (15-100 MBq) and left stirring for 5 minutes at room temperature. Each sample was then analyzed by silica gel TLC using 15 mM EDTA in 10% ammonium acetate/MeOH (50/50) as the mobile phase. Using this system, "free" ^{64}Cu has an $R_F = 0.66$, whereas $^{64}Cu(DTCBP)_2$ has a $R_F = 0.44$. Very efficient labeling (10 GBq/mg, radiochemical yield 100 %) was found when concentrations equal or higher to 1.5×10^{-4} M were used.

Alternatively, the radiolabeling of $^{64}Cu(DTCBP)_2$ can be analyzed using the same TLC system as above but using concentrations of EDTA up to 50 mM EDTA in 10% ammonium acetate/MeOH (50/50) as the mobile phase. Using 50 mM EDTA, "free" ^{64}Cu has an $R_F = 0.95$, whereas $^{64}Cu(DTCBP)_2$ retains a $R_F = 0.44$.

Stability of $^{64}Cu(II)(DTCBP)_2$.

Stability in PBS. To assess the *in vitro* stability of $^{64}Cu(DTCBP)_2$, 10 μL of ^{64}Cu $(DTCBP)_2$ (~1 MBq) were incubated in PBS (500 μL) in a 5% CO_2/95% air atmosphere at 37 ºC and constant shaking for 48 h. Aliquots (2 μL) were taken at 1, 24 and 48 h after mixing and analysed by TLC with silica gel plates using 15 mM EDTA in 10% Ammonium acetate/MeOH (50/50) as the mobile phase.

Stability in human serum. Human serum samples (500 μL) were incubated with 10 μL (~5 MBq) of $^{64}Cu(DTCBP)_2$ in a 5% CO_2/95% atmosphere. Aliquots (2 μL) were taken at 1, 24 and 48 h after mixing and analysed by TLC as above. 50 μL were also taken at the same time points and the proteins precipitated by addition of EtOH (70 μL). The samples were centrifuged and the supernatant separated. The precipitated proteins were washed twice with 70 μL of EtOH. The radioactivity of supernatant and precipitated proteins was counted using a capintec or a gamma counter.

To assess the strength of the serum protein-$^{64}Cu(DTCBP)_2$ binding, an identical experiment was setup but at each time point, instead of adding EtOH, 500 μg of HA were added. The tubes were then centrifuged for 5 min at 8609 rcf and the supernatant and HA pellet analyzed using a gamma counter.

To assess the strength of the serum protein-^{64}Cu binding, 5 MBq of ^{64}Cu were added to human serum and after 30 min. of constant shaking at 37 ºC, TLC analysis showed that ^{64}Cu migrated to $R_F = 0.66$.

Stability in acid (5 M HCl). $^{64}Cu(DTCBP)_2$ (20 μL, 5 MBq) was added to 200 μL of 5M HCl and the mixture heated to 60 ºC. After 5 min, TLC showed that $^{64}Cu(II)(DTCBP)_2$ had fully decomposed.

Stability in EDTA solutions at pH 4. To assess the *in vitro* stability of $^{64}Cu(DTCBP)_2$, 20 μL of ^{64}Cu $(DTCBP)_2$ (~5 MBq) were incubated in 100 μL of a EDTA solution of different concentrations at pH 4 (100 mM acetate buffer) and incubated at 25 ºC. Aliquots (2 μL) were taken at 1, 5, 10 and 24 h after mixing and analysed by TLC with silica gel plates using 15 mM EDTA in 10% Ammonium acetate/MeOH (50/50) as the mobile phase. $^{64}Cu(DOTA)$ was also synthesized following standard procedures and assessed

using the same procedure as with $^{64}Cu(DTCBP)_2$, but using 10% Ammonium acetate/MeOH (50/50) as the mobile phase for the TLC studies.

The results are summarized below:

- Incubation in 3 mM (1 mg/mL) EDTA at pH 4, 25 ºC:
    - $^{64}Cu(DTCBP)_2$ – 100 % intact for 5 h (22% intact at 10 h)
    - ^{64}Cu (DOTA) – 100 % intact (24 h)
- Incubation in 10 mM EDTA at pH 4, 25 ºC:
    - $^{64}Cu(DTCBP)_2$ – 100 % intact for 1 h (100%) (20% intact at 5 h)
    - ^{64}Cu(DOTA) – 100 % intact (24 h)
- Incubation in 100 mM EDTA at pH 4, 25 ºC:
    - $^{64}Cu(DTCBP)_2$ –100 % decomposition by 1 h
    - ^{64}Cu(DOTA) – 100 % intact at 24 h

***In vitro* inorganic salt binding study.**

In order to assess the binding of $^{64}Cu(DTCBP)_2$ to different inorganic salts, 1 mg/mL suspensions of gadolinium oxide (Gd_2O_3), hydroxyapatite (HA), europium oxide (Eu_2O_3), erbium oxide (Er_2O_3), iron oxide (Fe_3O_4), indium oxide (In_2O_3), calcium carbonate (CC), Ytterbium oxide (Yb_2O_3), aluminium oxide (Al_2O_3), calcium phosphate (CP), titanium oxide (TiO_2), β-tricalcium phosphate (b-CP); calcium pyrophosphate (CPy), tin oxide (SnO), silicon oxide (SiO_2) and calcium oxalate (CO) in 50 mM TRIS pH 6.9 were prepared in triplicate. Immediately after, 500 kBq of $^{64}Cu(DTCBP)_2$ were added to each suspension and vortexed for 1 h at room temperature. The solutions were

then centrifuged (5 min, 20238 rcf) and a 100 μL aliquot of the supernatant of each sample analyzed using a gamma counter. Control samples lacking salt were also prepared and counted as a control. The results are expressed as % binding using the following equation:

$$\%\ binding = \left(1 - \frac{CPM_s}{CPM_c}\right) x100$$

where CPM_s are the counts per minute of each sample and CPM_c are the counts per minute of the control.

Synthesis of ^{64}Cu(DTCBP)$_2$-Endorem. 20 μL of Endorem (224 μg Fe) were added to a 100 μL solution of ^{64}Cu(DTCBP)$_2$ and the vial sealed and heated for 100ºC for 15 min. The vial was then cooled in an ice bath and the contents transferred to a Vivaspin 500 filter with a 10 KDa molecular weight cut-off membrane that had been previously washed with 3 × 500 μL of H_2O. The filter was centrifuged at 8609 rcf for 3 min, separating approximately 50 μL of retentate that contained the radiolabeled nanoparticles from 450 μL of filtrate that contained most of the unbound ^{64}Cu(DTCBP)$_2$. To the retentate was added 100 μL of saline and the mixture sonicated for 1 min and centrifuged as before. This process was repeated 5 times until no more unbound ^{64}Cu(DTCBP)$_2$ was detected in the filtrates. The total radioactivity in the filtrates and retentates was measured to determine the radiolabeling yield (%). The retentate solution containing ^{64}Cu(DTCBP)$_2$-Endorem was then made up to 100 μL by adding saline, mixed, removed from the Vivaspin filter and filter-sterilized using a 0.2 μm hydrophilic PTFE filter (13 mm

diameter). This step was necessary for the *in vivo* studies in order to avoid potential dust particles and bacteria that may compromise the wellbeing of the animals after i.v. injection of the agent. Less than 5% total radioactivity was retained in both filters (10 kDa size-exclusion and sterilizing), meaning that, if any, less than 5% of the nanoparticles were present as aggregates bigger than 220 nm after the labeling process.

In order to discard the possibility that $^{64}Cu(DTCBP)_2$ reacts or gets trapped inside the dextran coating of Endorem we prepared a concentrated solution of dextran (40 kDa, 9 mM) and added a solution of $^{64}Cu(DTCBP)_2$ (5 MBq), followed by 15 min heating at 100 ºC. These conditions were chosen in order to mimic the radiolabeling conditions used and high concentration of the dextran coating around the Fe_3O_4 cores of Endorem. After cooling the mixture to room temperature, it was transferred to a 10 KDa centrifugal filter, centrifuged, and the retentate washed several times with saline (NOTE: The absence of dextran from the filtrate was confirmed using the 4% phenol/sulfuric acid assay and by the lack of characteristic dextran vibrations in the IR spectrum of the filtrate). $^{64}Cu(DTCBP)_2$ quantitatively eluted from the filter, separating from the dextran solution. Increasing the reaction time had no effect in the result. However, the same experiment was repeated in the presence of uncoated SPIOs (300 μg), resulting in 100% of $^{64}Cu(DTCBP)_2$ being retained in the filter and hence bound to the SPIO-dextran mixture. Thus, these experiments strongly suggest that $^{64}Cu(DTCBP)_2$ is not simply buried within the dextran coating in $^{64}Cu(DTCBP)_2$-Endorem but instead binds through its BP groups to the surface of the metal core of Endorem.

We have also examined an alternative method for the synthesis of $^{64}Cu(DTCBP)_2$-Endorem by reacting DTCBP with Endorem in the same manner as above, followed by

purifying the DTCBP-Endorem complex using a 10 kDa filter, and adding ^{64}Cu at the last step without heating, followed by washings with 10 kDa MWCO filter. This method results in lower radiochemical yields of 20%. This is not surprising because if DTCBP binds to the nanoparticle before the metal complex is formed, the DTCBP ligands may be too far apart in the nanoparticle surface to form the desired bis-dithiocarbamate complex. It is therefore more likely that the complex seen consists of one dithiocarbamate coordinated to the metal centre with the remaining coordination sites being occupied by donor atoms from Endorem. The exact identity of the copper complex formed, however, can not be ascertained using this method. (NOTE: Reaction of free ^{64}Cu with Endorem results in 0% binding after several washings of the Endorem-^{64}Cu mixture with a 10 kDa MWCO filter, hence the presence of DTCBP is essential for copper binding).

***In vitro* stability studies of $^{64}Cu(DTCBP)_2$-Endorem in PBS and human serum**. To assess the *in vitro* stability of $^{64}Cu(DTCBP)_2$-Endorem, triplicate experiments with 10 μL of a solution of this compound were incubated in PBS (500 μL) and human serum (500 μL) in a 5% CO_2/95% air atmosphere at 37 ºC and constant shaking for 48 h. Aliquots of 250 μL were taken at 1, 24 and 48 h after mixing and these were centrifuged at 20238 rcf for 15 min. The radioactivity of the pellets and supernatants was measured to give the percentage of radioactivity bound to the nanoparticles. After the measurement, the supernatant and pellet were mixed with the remaining sample and returned to the incubator.

Alternatively, the *in vitro* stability of $^{64}Cu(DTCBP)_2$-Endorem in serum can also be assessed using a 100 kDa molecular weight cutoff filter. The results obtained are

consistent with the previous method, with 100 % of the radioactivity being retained in the retentate and hence bound to the nanoparticles for at least 30 h.

***In vitro* stability studies of ^{64}Cu(DTCBP)$_2$-Endorem in 10 mM EDTA at pH 4.**

To assess the *in vitro* stability of ^{64}Cu(DTCBP)$_2$-Endorem, 20 μL of ^{64}Cu(DTCBP)$_2$-Endorem (15 MBq) were incubated in 100 μL of a 10 mM EDTA solution at pH 4 (100 mM acetate buffer) and incubated at 25 ºC. Aliquots (2 μL) were taken at 1, 5, 10 and 24 h after mixing and analysed by TLC with silica gel plates using 15 mM EDTA in 10% Ammonium acetate/MeOH (50/50) as the mobile phase. The radioactivity and iron oxide remained at the origin of the TLC plate throughout the experiment.

TEM and DLS studies of ^{64}Cu(DTCBP)$_2$-Endorem.

Samples of ^{64}Cu(DTCBP)-Endorem (decayed) and Endorem were analyzed by TEM (Fig. S4) and DLS (Fig. S5) to confirm that the radiolabeling step had not modified the size of the SPIO nanoparticles significantly. Thus, the TEM images confirm that the Fe_3O_4 cores of ^{64}Cu(DTCBP)-Endorem remain unaltered with an average size of 5 nm. DLS studies confirm that there is a slight decrease in hydrodynamic size (108 ± 60 nm , compared to 127 ± 60 nm for Endorem). This small decrease in hydrodynamic size is consistent with previous results and is the result of the heating step during radiolabeling.[6]

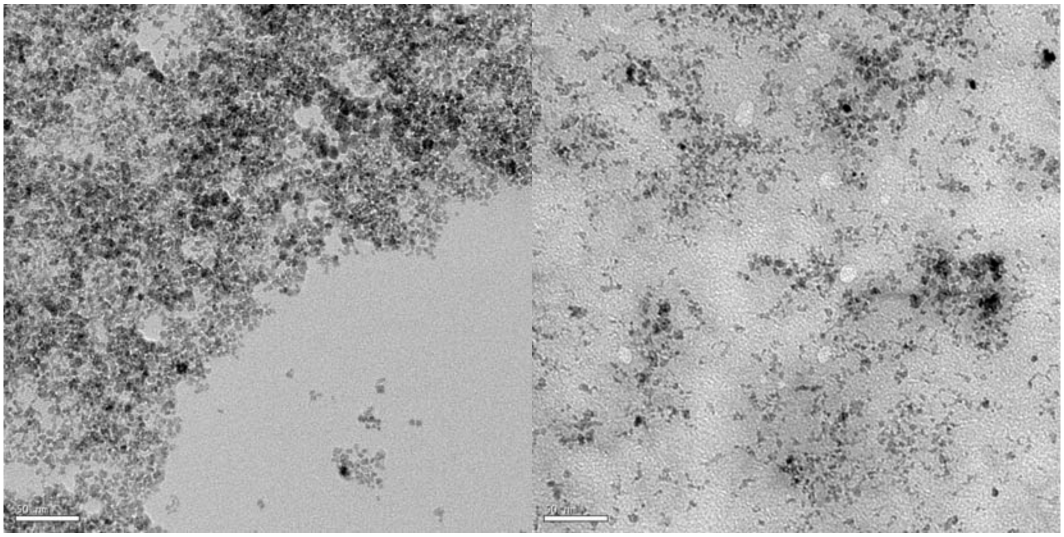

Fig S4. TEM images of Endorem (left) and ^{64}Cu(DTCBP)-Endorem (decayed, right) showing the 5 nm Fe_3O_4 cores of the nanoparticles.

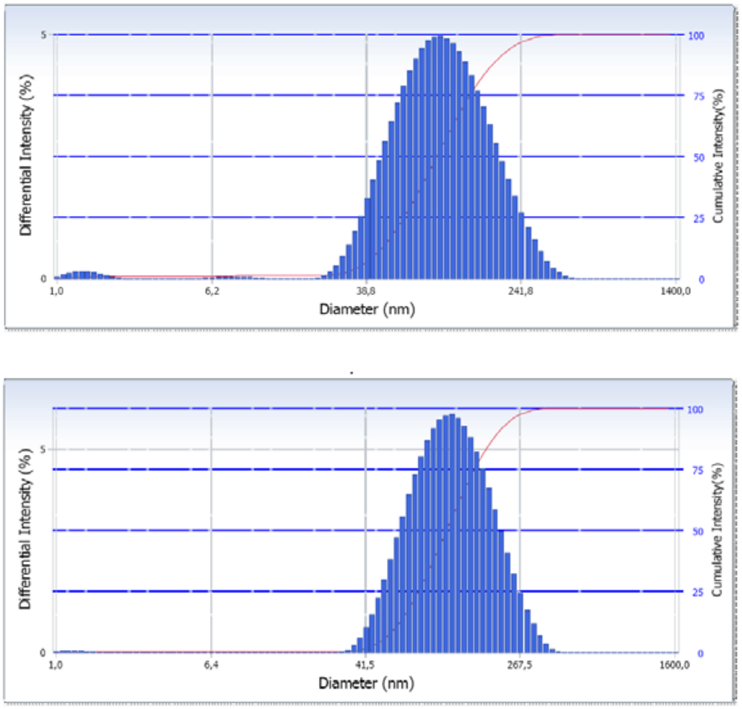

Fig S5. DLS measurements of ^{64}Cu(DTCBP)-Endorem (decayed) (top, 108 ± 60 nm) and Endorem (bottom, 127 ± 60 nm)

MR and PET-CT imaging:

Magnetic Resonance Imaging (MRI) scanner. MR imaging was performed on a wide-bore vertical 400 MHz (9.4 T) MR scanner (Bruker Avance III, Germany). The gradient coil had an inner diameter of 40 mm and gradient strength was 800 mT/m. A quadrature transmit/receive coil with an internal diameter of 35 mm was used. The acquisition time was 4 ± 0.5 minutes. Temperature was maintained at 37 ºC using warm water running through the gradient coil.

PET-CT scanning. PET images were acquired with a NanoPET-CT™ preclinical animal scanner (Mediso Ltd., Bioscan Inc.). Image acquisition took place in list mode format. Acquisition time was 30 min. List-mode data were sorted into 400-600 keV energy-window, 5 ns time-window; crystal-efficiency correction was applied. Reconstruction method was OSEM (6 subsets, 8 iterations, 0.29 mm pixel size, 0.585 mm axial thickness) based on SSRB 2D LOR rebinning (linear interpolation, 16 span-size). The CT images were obtained with 55 kVp tube voltage, 1200 ms exposure time in 360 projections. The two modalities (PET and CT) were fused using InVivoScope (Bioscan) software.

***T_2* relaxation measurement *in vitro*.** Samples of ^{64}Cu(DTCBP)-Endorem (decayed) and Endorem were prepared with Fe concentrations of 23, 18, 9, 1.8 and 0.37 mM by serial dilution, along with a control sample (0.00 mM Fe). For comparison a multiple echo spin-echo sequence was acquired with the following sequence: FOV = 40 × 40 mm^2; resolution = 0.156 × 0.156 mm^2; slice thickness = 1.5 mm; deltaTE/TR = 10/2000 ms and 16 echoes. Quantitative parameter maps of the transverse R_2 relaxation rate were produced using the image data acquired from multiple echo times (TEs). The signal,

S(TE) at each pixel was fitted to a model for mono-exponential decay with TE:S(TE) = $S_0 \cdot \exp(-R_2 \cdot TE)$, where S_0 represents the signal intensity at TE = 0 ms and $R_2 = 1/T_2$. The average R_2 value within each sample was calculated using regions of interest in the parameter maps and plotted against the Fe concentration of each sample. R_2 (ms^{-1}) was calculated for each concentration of both compounds and these were plotted against iron concentration. The data points obtained were then fitted to a straight line and the slope (relaxivity r_2) calculated (Figure 3A). The r_2 value obtained for ^{64}Cu(DTCBP)-Endorem was 34 s^{-1} mM^{-1} whereas for Endorem was 30 s^{-1} mM^{-1}.

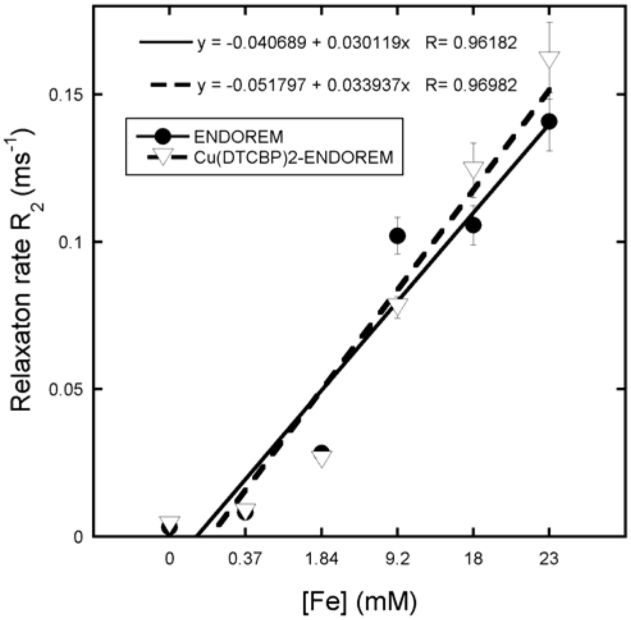

***In vivo* MR and PET-CT imaging.** The *in vivo* imaging procedure was carried out in accordance with British Home Office regulations governing animal experimentation and performed as follows. A six-week-old female C57BL/6 mouse was anesthetized by subcutaneous injection of 200 μL of a mixture of 10% Dormitor (medetomidine) and

6.8% Vetalar (ketamine hydrochloride) in saline. After 15 min the mouse was placed in prone position on the MRI mouse bed and a pressure-transducer was placed under the mouse abdomen for respiratory monitoring. Typical respiration rates were between 40 ± 10 min^{-1} (1500 ± 500 ms per respiratory cycle). Cine-FLASH was used to acquire temporally resolved dynamic short-axis MR images of the popliteal lymph nodes. Spoiler gradients of 1 ms duration and 100 mT/m strength were applied after each data acquisition readout to dephase the remaining transverse magnetization before the application of the next radio frequency (RF) excitation pulse. Cine-FLASH T_2*-weighted was performed without the use of triggering. Imaging parameters were as follows: repetition time (TR) = 500 ms; echo time (TE) = 5 ms; field of view (FOV) = 30 x 30 mm; matrix size = 128 ×128; slice thickness = 1 mm; flip angle = 20º; 3 averages, 5 slices, 1 frame. Short axis and coronal views of the popliteal lymph nodes were acquired. The mouse was then removed from the MRI scanner and injected subcutaneously a 20 µL solution of $^{64}Cu(II)(DTCBP)_2$-Endorem in saline (2 MBq, 44 µg Fe) via the footpad. After 3 h, the mouse was injected with another 100 µL of the anesthetic mixture and the same MRI scans as described above were repeated, followed by a PET-CT scan in the NanoPET-CT™ scanner.

References

[1] J. C. Dittmer, R. L. Lester, *J. Lipid Res.* **1964**, *5*, 126.
[2] H. E. Gottlieb, V. Kotlyar, A. Nudelman, *J. Org. Chem.* **1997**, *62*, 7512.
[3] Y. S. Kang, S. Risbud, J. F. Rabolt, P. Stroeve, *Chem. Mater.* **1996**, *8*, 2209.
[4] D. W. McCarthy, R. E. Shefer, R. E. Klinkowstein, L. A. Bass, W. H. Margeneau, C. S. Cutler, C. J. Anderson, M. J. Welch, *Nuc. Med. Biol.* **1997**, *24*, 35.
[5] A. Lombardi, D. Marasco, O. Maglio, L. Di Costanzo, F. Nastri, V. Pavone, *Proc. Natl. Acad. Sci. U S A* **2000**, *97*, 11922.

[6] R. Torres Martin de Rosales, R. Tavaré, A. Glaria, G. Varma, A. Protti, P. J. Blower, *Bioconjugate Chem.* **2011**, DOI: 10.1021/bc100483k.
